# Case report: Prenatal diagnosis in the fetus of a couple with both thalassemia and deafness genes

**DOI:** 10.3389/fgene.2023.1258293

**Published:** 2023-12-11

**Authors:** Youqiong Li, Liang Liang, Jinping Bai, Lihong Zheng, Ting Qin

**Affiliations:** Center for Medical Genetics and Prenatal Diagnosis, People’s Hospital of Guangxi Zhuang Autonomous Region, Nanning, Guangxi, China

**Keywords:** thalassemia, deafness, hearing loss (HL), prenatal screening, expanded carrier screening (ECS)

## Abstract

**Background:** Prenatal diagnosis and genetic counseling play an important role in preventing and controlling birth defects. No reports were found of prenatal diagnosis of couples carrying both the thalassemia and deafness genes. In this study, we presented the prenatal screening and diagnosis of a couple with both thalassemia and deafness genes, contributing to better genetic counseling.

**Case Report:** A couple visited our hospital for a routine prenatal examination. As required by the policy in our region, they underwent screening and genetic diagnosis for thalassemia. Meanwhile, they did not accept the recommendation to test for spinal muscular atrophy and deafness genes. The female was confirmed to be a Hb Quong Sze (Hb QS) carrier (αQSα/αα, βN/βN), and the male had Hb H disease combined with β-thalassemia (--SEA/αCSα, βCDs41-42 (-TTCT)/βN). A prenatal diagnosis of the fetus revealed a Hb CS heterozygote. Subsequent complementary testing showed that the male was a double heterozygote of the *GJB2* gene c.299_300delAT combined with c.109G>A, and Sanger sequencing confirmed that the female was a carrier of c.508_511dup in the *GJB2*. Fortunately, the chorionic villi results indicated that the fetus was only a carrier of deafness.

**Conclusion:** Since both partners carried thalassemia and deafness genes, the couple required prenatal diagnosis for the respective mutations. Expanded carrier screening (ECS) is a more advanced technology that can detect multiple disease genes simultaneously.

## Introduction

Alpha-thalassemia is a group of autosomal recessive hemoglobinopathies caused by a failure to synthesize one or more hemoglobin chains (Baird et al., 2022). The more severe form of α-thalassemia is hemoglobin Bart’s hydrops fetalis (Hb Bart) syndrome, in which an enlarged placenta, marked hepatosplenomegaly, generalized edema, and effusion can be found during pregnancy (Horvei et al., 2021). Even if they survive until birth, death usually occurs. In contrast, β-thalassemia major needs lifelong blood transfusions starting early in childhood (usually before age two). Exactly, it is not only an essential public health problem but also an economic burden for many countries in the region with a high prevalence of thalassemia. For this reason, in Guangxi, a region with a high prevalence of thalassemia, free thalassemia screening has become a government-promoted policy, and the population thalassemia screening rate has exceeded 98% (Chen, et al., 2022).

Hearing loss (HL) is a frequent sensory defect in humans, and according to the World Health Organization, more than 5% of the world’s population (approximately 430 million) suffered from HL by 2021 (Mao, et al., 2023). Gene mutations are the main contributors to HL. HL can significantly impact child development, including speech acquisition and cognitive, social, and emotional development. However, in China, molecular screening and diagnosis of the HL gene are mainly used for newborn screening and are rarely performed prenatally. There are no reports of prenatal diagnosis in couples carrying both thalassemia and deafness gene mutations. In the study, we describe a couple carrying the genes for thalassemia and deafness that need prenatal diagnosis to avoid having a child with severe thalassemia and deafness.

## Case report

**FIGURE 1:**
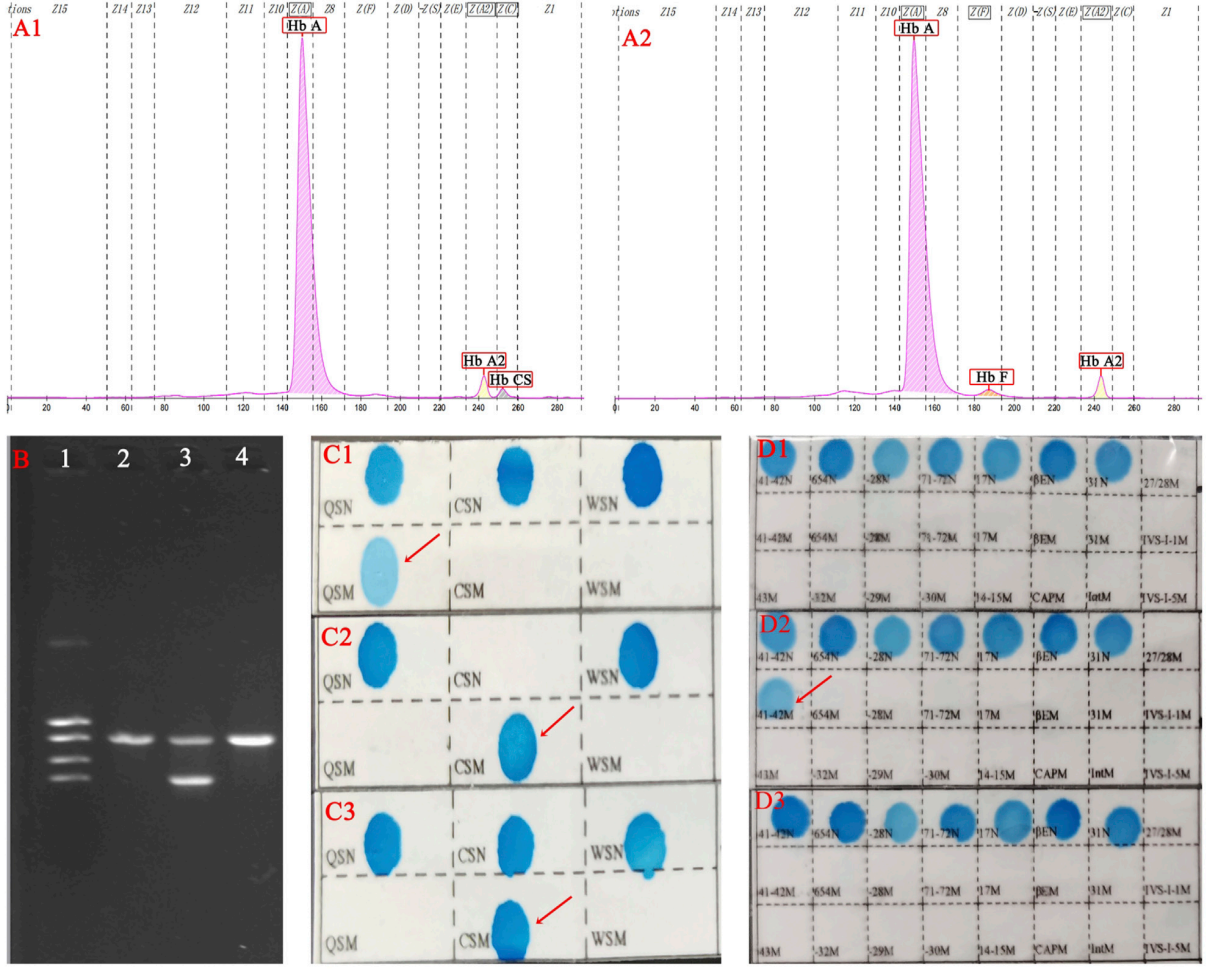
Hemoglobin analysis and molecular diagnosis in this family. The male (--^SEA^/α^CS^α, β^CDs41-42(−TTCT)^/β^N^) CE result showed Hb CS but no Hb H because of the combination of β-thalassemia **(A1)**. The female (α^QS^α/αα, β^N^/β^N^) was a Hb QS carrier, but there was no abnormal finding on the CE program because Hb QS was not detected by CE **(A2)**. Agarose gel electrophoresis **(B)** suggested normal for the female (1), SEA heterozygous state for the male (2), and normal for the fetus (3). The female was heterozygous for Hb QS **(C1)**, the male was homozygous for Hb CS **(C2)**, and the fetus was heterozygous for Hb CS **(C3)** in the α-globin chain using reverse dot blot hybridization (RDB). Normal was found in female **(D1)** and fetus **(D3)**, but CDs41-42 (-TCTT) were heterozygous state in male **(D2)** by RDB.

A couple (28 years old for the female and 30 years old for the male) were referred to our hospital for a pregnancy examination. They were required to undergo free screening for thalassemia according to the thalassemia prevention and control policy in the Guangxi region. The doctor also recommended that they undergo genetic testing for deafness and spinal muscular atrophy (SMA), but they did not approve, probably because of the cost of the tests. The hematological indices for female and male were 70.8 fL and 58 fL for mean corpuscular volume (MCV) (reference: 82.0–100 fL), 22.2 pg and 16.1 pg for mean corpuscular hemoglobin (MCH) (reference: 27–31 pg). Hemoglobin analysis showed Hb A 95.7%, Hb F 1.5%, and Hb A_2_ 2.8% in the female, and Hb A 95.8%, Hb A_2_ 2.9%, and Hb Constant spring (Hb CS) 1.3% in the male using capillary electrophoresis (CE) (Capillary 2 Flex Piercing; Sebia, Lisses, France). Gap-PCR and reverse dot blot hybridization revealed an α^QS^α/αα genotype in the female and a--^SEA^/α^CS^α and β^CDs41-42(−TTCT)^/β^N^genotype in the male (Yaneng Ltd., Shenzhen, Guangdong, China) (Figure 1).

**FIGURE 2:**
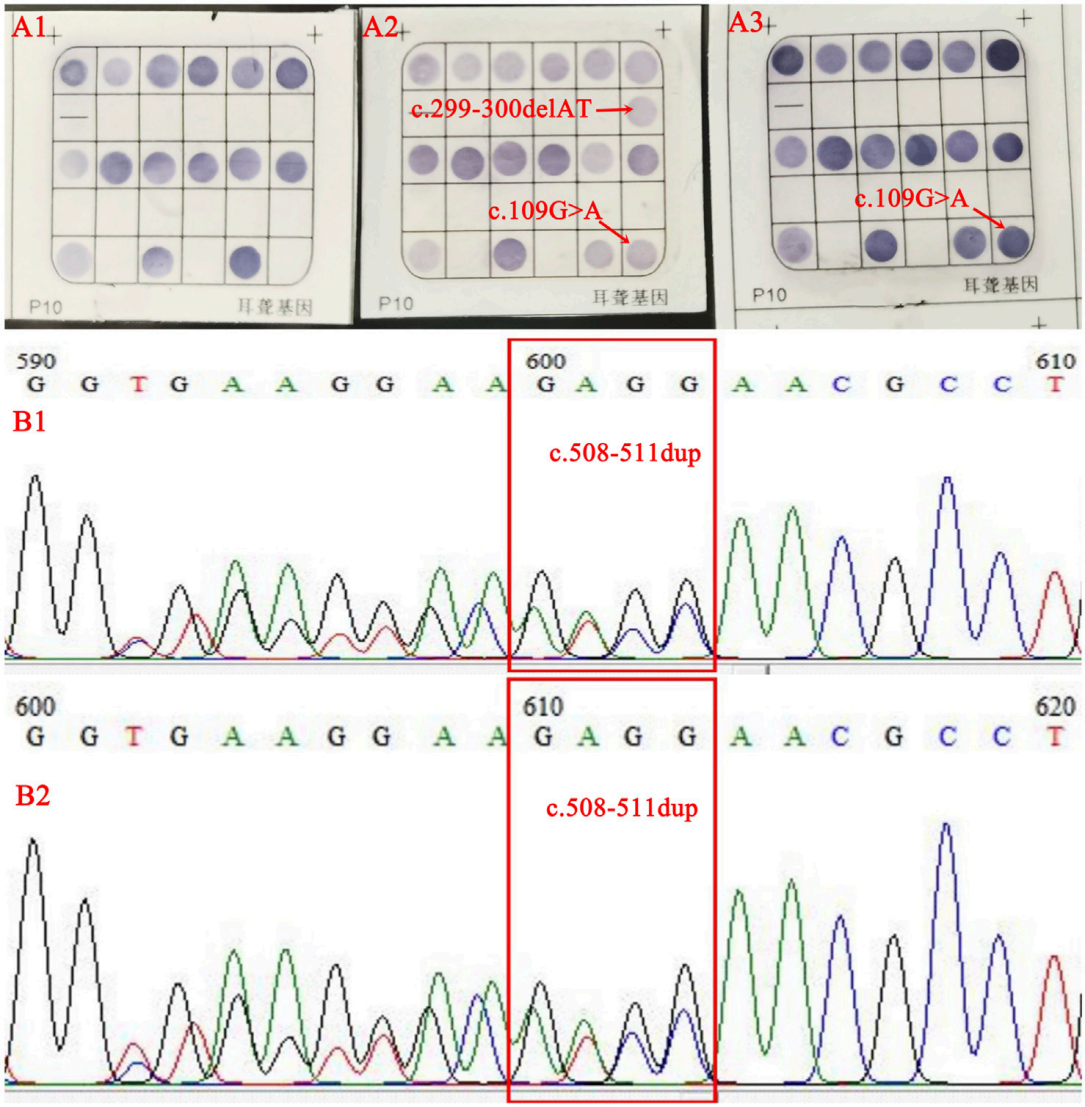
The results of flow-through hybridization were normal in the female **(A1)**, compound heterozygosity for c.299_300delAT and c.109G>A in the male **(A2)**, and a c.109G>A heterozygous **(A3)** in the fetus. Sanger sequencing identified a c.508_511dup heterozygous state in female **(B1)** and fetus **(B2)**.

Since a patient with--^SEA^/α^CS^α or--^SEA^/α^QS^α may sometimes require transfusion or even dependent on this therapy, the couple was advised to carry out a prenatal diagnosis of the thalassemia (Jiang, et al., 2020). The interventional puncture was performed to collect chorionic villi, and the molecular result was αα/α^CS^α after obtaining informed consent from the pregnant female. The couple was delighted when the doctor explained that they would not give birth to a child with severe thalassemia. They agreed to be tested at this genetic counseling when their doctor again suggested testing for SMA and deafness genes. Genetic testing for deafness displayed a double heterozygote of the *GJB2* gene c.299_300delAT combined with c.109G>A in the male and no abnormalities in the female by flow-through hybridization (Chaozhou Hybribio Limited Corporation, Chaozhou, China). Because the male was the carrier of the deafness gene mutation, we had to sequence the *GJB2* gene in the female to rule out the possibility of mutations outside the detection range. Surprisingly, the female was also found to be a carrier with c.508_511dup in the *GJB2*. So far, we have determined that the couples carry both the thalassemia and deafness gene mutations. The prenatal diagnosis of deafness in the villi was c.508_511dup combined with c.109G>A in the *GJB2* gene (Figure 2).

## Discussion

The *GJB2* gene is the most common HL gene globally, and its mutations explain up to 50% of non-syndromic deafness. The Human Gene Mutation Database (https://www.hgmd.cf.ac.uk/ac/index.php) has compiled over 300 *GJB2* missense variants, but only about 100 missense variants have a proven function, and two-thirds of the functions remain to be investigated. The c.299_300delAT mutation is considered pathogenic, whereas the pathogenicity of c.109G>A is debated (Riza, et al., 2022). The male had no hearing abnormalities, and the female did not complain of hearing loss in this study. However, after completing a prenatal diagnosis of thalassemia, the couple’s deafness gene supplementation test revealed that they both carried the deafness gene. In particular, the female harbored a rare mutation (c.508_511dup) that was not within the detection range. This suggested that the couple was at risk of having a child with deafness in addition to a child with severe types of thalassemia. By reviewing the literature, there are no reports of couples carrying different monogenic disease mutations simultaneously for prenatal diagnosis.

The chorionic villi had to be supplemented with a prenatal molecular diagnosis of deafness. Fortunately, the fetus inherited the c.508_511dup from the female and the c.109G>A from the male. It should have a similar phenotype to the male and can continue her pregnancy. After more than 10 years of efforts in Guangxi, the people understand and are aware of thalassemia. However, there is still a lack of knowledge about deafness, SMA, and other diseases. This case focused on thalassemia and ignored the deafness, almost putting them at risk of having a child with deafness. It cautions us to be aware of the possibility of carrying multiple genetic disease risks in our daily clinical practice. Therefore, given the limitations of single-item testing and the time-consuming cost of combined testing of various items, there is a clinical expectation for a test that can simultaneously detect multiple diseases.

Developments in high-throughput technologies, for example, next-generation sequencing (NGS) and third-generation sequencing (TGS), enable the simultaneous study of multiple disease genes in a time- and cost-efficient way, allowing carrier screening to move toward a more comprehensive and broad approach, i.e., expanded carrier screening (ECS). It has been applied to screen carriers of thalassemia, hereditary deafness, Wilson’s disease, and phenylketonuria (Shi, et al., 2021; Fang, et al., 2023). If couples undergo ECS prior to conception, they can receive a comprehensive assessment of their genetic risk and make an informed decision to have healthy offspring. Despite its advantages, there are some limitations. The main limitation of using NGS and TGS in ECS is that their costs remain high. In addition, some weaknesses are also present, such as the creation of unexpected information, uncertainty about the phenotype of a disease-specific condition for which an individual may be a carrier, and the need for further confirmatory testing (Sparks, 2020; Veneruso, et al., 2022). Therefore, all clinical laboratories in China are first advised to select the most familiar and economical technique for routine use. The use of NGS or TGS for re-checking is only to be suggested when there is a failure to identify by routine methods. In this study, prenatal diagnostic results were accurately obtained using ECS, thus avoiding the risk of missed diagnoses.

## Data Availability

The original contributions presented in the study are included in the article/supplementary material, further inquiries can be directed to the corresponding author.

## References

[B1] Baird D. C. Batten S. H. Sparks S. K. (2022). Alpha- and beta-thalassemia: rapid evidence review. Am. Fam. Physician. 105, 272–280. 35289581

[B2] Chen P. Lin W. X. Li S. Q. (2022). THALASSEMIA in ASIA 2021: thalassemia in Guangxi province, People’s Republic of China. Hemoglobin 46, 33–35. 10.1080/03630269.2021.2008960 35950576

[B3] Fang Y. Li J. Zhang M. Cheng Y. Wang C. Zhu J. (2023). Clinical application value of expanded carrier screening in the population of childbearing age. Eur. J. Med. Res. 28, 151. 10.1186/s40001-023-01112-8 37031186 PMC10082524

[B4] Horvei P. MacKenzie T. Kharbanda S. (2021). Advances in the management of α-thalassemia major: reasons to be optimistic. Hematol. Am. Soc. Hematol. Educ. Program 2021, 592–599. 10.1182/hematology.2021000295 PMC8791144 34889445

[B5] Jiang F. Xu L. L. Chen G. L. Zhou J. Y. Li J. Tang X. W. (2020). Hematological characteristics of Hb constant spring (HBA2: c.427T>C) carriers in mainland China. Hemoglobin 44, 86–88. 10.1080/03630269.2020.1755979 32338097

[B6] Mao L. Wang Y. An L. Zeng B. P. Wang Y. Y. Frishman D. (2023). Molecular mechanisms and clinical phenotypes of *GJB2* missense variants. Biol. (Basel). 12, 505. 10.3390/biology12040505 PMC10135792 37106706

[B7] Riza A. L. Alkhzouz C. Farcaș M. Pîrvu A. Miclea D. Mihuț G. (2022). Non-syndromic hearing loss in a Romanian population: carrier status and frequent variants in the *GJB2* gene. Genes (Basel). 14, 69. 10.3390/genes14010069 36672810 PMC9858611

[B8] Shi M. Liauw A. L. Tong S. Zheng Y. Leung T. Y. Chong S. C. (2021). Clinical implementation of expanded carrier screening in pregnant women at early gestational weeks: a Chinese cohort study. Genes(Basel). 12, 496. 10.3390/genes12040496 33805278 PMC8066122

[B9] Sparks T. N. (2020). Expanded carrier screening: counseling and considerations. Hum. Genet. 139, 1131–1139. 10.1007/s00439-019-02080-y 31679051 PMC7195224

[B10] Veneruso I. Di R. C. Tomaiuolo R. D'Argenio V. (2022). Current updates on expanded carrier screening: new insights in the omics era. Med. Kaunas. 58, 455. 10.3390/medicina58030455 PMC8951681 35334631

